# Effects of Mechanical Stress on Bone and Cartilage Metabolism: How Mechanical Stress Affects Energy Metabolism in Bone and Cartilage Tissues (Our Research Overview): Mini Review

**DOI:** 10.3390/ijms27031380

**Published:** 2026-01-30

**Authors:** Hideaki Iwata, Satomi Sato, Shu Somemura, Masahiro Takemoto, Yuki Takahashi-Suzuki, Yodo Sugishita, Hiroto Fujiya, Naoki Haraguchi, Kazuo Yudoh

**Affiliations:** 1 Department of Orthopaedic Surgery, St. Marianna University School of Medicine, Kawasaki 216-8511, Japan; hideaki.iwata@marianna-u.ac.jp (H.I.); satomi.kimura@marianna-u.ac.jp (S.S.); masahiro.takemoto@marianna-u.ac.jp (M.T.); naoki.haraguchi@marianna-u.ac.jp (N.H.); 2 Department of Sports Medicine, St. Marianna University School of Medicine, Kawasaki 216-8512, Japan; s3somemura@marianna-u.ac.jp (S.S.); fujiya-1487@marianna-u.ac.jp (H.F.); 3 Department of Frontier Medicine, Institute of Medical Science, St. Marianna University School of Medicine, Kawasaki 216-8511, Japan; y2takahashi@marianna-u.ac.jp (Y.T.-S.); yodo@marianna-u.ac.jp (Y.S.)

**Keywords:** mechanical stress, bone metabolism, cartilage metabolism, osteoblast, chondrocyte, energy metabolism, mitochondria

## Abstract

Bone resorption and formation are known to change in response to mechanical stress. The mechano-transduction mechanism by which bone tissue senses the stress, altering cellular activity in response via intracellular signaling pathways, ultimately leading to physiological and pathological changes, is beginning to be elucidated. Furthermore, excessive mechanical stress on bone and joints due to aging, obesity, overload, and overuse is thought to cause decreased chondrocyte activity, degeneration and destruction of the cartilage collagen matrix, degeneration of the subchondral bone, and joint dysfunction, contributing to the progression of osteoarthritis (OA). However, much remains unknown about how osteoblasts, responsible for bone formation, and chondrocytes, responsible for cartilage homeostasis, sense and respond to mechanical stress. Furthermore, whether there are mechanisms to protect against pathological and excessive mechanical stress in bone and cartilage tissue, their associated molecular mechanisms, and the relationship between mechanical stress responses and osteochondral degeneration, remain unknown. Understanding these mechanisms is considered essential for the development of new therapeutic strategies for osteochondral diseases. Our research aims to deepen our understanding of the etiology and pathophysiology of bone and cartilage diseases (osteoporosis, fragility fracture, and OA) and to develop new treatments from the perspective of mechanical stress response. In this paper we review the latest findings regarding the roles of cellular energy regulators (glucose transporters and energy sensors) and mechanical stress response factors, and the relationship between these factor-mediated changes in energy metabolism and osteochondral degeneration. This minireview discusses how energy metabolism regulators control the activity of both osteoblasts and chondrocytes in osteochondral tissue in response to mechanical stress.

## 1. Introduction

In an aging society, the number of elderly people suffering from age-related osteochondral diseases such as osteoporosis, fragility fractures, and osteoarthritis (OA) is rapidly increasing, leading to a sharp rise in the rate of bedridden patients [1,2]. Osteoporosis is a global problem with a global prevalence of 18.3% and concomitant fragility fractures can reduce survival by approximately 20% [3,4,5]. In Japan, the prevalence of osteoporosis is estimated to be about 15.0 million, which accounts for approximately 10% of the total population, and approximately 193,400 hip fractures occur annually [6,7,8]. OA is a progressive degenerative joint disease based on the degeneration and wear of articular cartilage, secondary synovitis, and new bone and cartilage formation, leading to impaired daily activities and reduced exercise capacity with age [9,10,11]. In Japan, it is estimated that there are over 25 million patients with radiographically diagnosed OA (showing joint deformation on X-ray), and over 12.8 million patients who experience symptoms such as joint pain and functional impairment, with a prevalence rate approximately 10 to 20 times higher than that of rheumatoid arthritis [11,12,13]. There are over 900,000 new OA cases annually in Japan, and the economic loss is estimated to reach 1% of the gross national product, with concerns about a further increase in the number of patients in the future [12,13,14]. In Japan, where the population is rapidly aging, measures to prevent people from becoming bedridden and to extend healthy life expectancy are urgently needed. In order to advance the prevention and treatment of osteoporosis, fragility fractures, and OA in the elderly, further studies are needed into the control mechanisms of bone and cartilage metabolism and therapeutic strategies for age-related osteochondral disorders.

It is well known that bone and cartilage metabolism changes in response to mechanical stress. The mechanisms underlying mechanical stress responses, which induce physiological and pathological changes in cellular activity via intracellular signaling pathways in osteoblasts, responsible for bone formation, and chondrocytes, responsible for maintaining cartilage homeostasis, are beginning to be elucidated [15,16,17]. However, how osteoblasts/chondrocytes sense and respond to mechanical stress remains unknown. Many questions remain, such as the differences between the response mechanisms to physiological mechanical stress and overload stress, and whether there are protective mechanisms against pathological overload stress. Further research is needed to clarify these questions. We have previously demonstrated that sirtuin 1 (SIRT1), which functions as a cellular energy regulator (energy sensor) in chondrocytes, is activated in response to mechanical stress [18]. Furthermore, we have shown that SIRT1 regulates the expression of runt-related transcription factor (Runx) 2, a transcription factor involved in hypertrophic cartilage formation and osteogenesis, and thereby regulates osteophyte formation and endochondral ossification [18]. Regarding the relationship between bone metabolism and mechanical stress, we have confirmed that mechanical stress in osteoblasts increases intracellular glucose uptake via activation of plasma membrane glucose transporter 1 (Glut1) [15]. We have also shown that this change reduces the activity of the energy sensor SIRT1, thereby enhancing the expression of Runx2, which is negatively regulated by SIRT1. These results indicate that mechanical stress may induce osteoblast activity via the Glut1–SIRT1–Runx2 pathway and accelerate osteogenic potential in bone metabolism [15].

Based on the results of our previous studies, this review provides an overview of the mechanisms underlying bone formation and cartilage degeneration in response to mechanical stress from the perspective of regulating cellular energy metabolism.

## 2. Molecular Events Caused by Mechanical Stress in Bone

**Figure 1:**
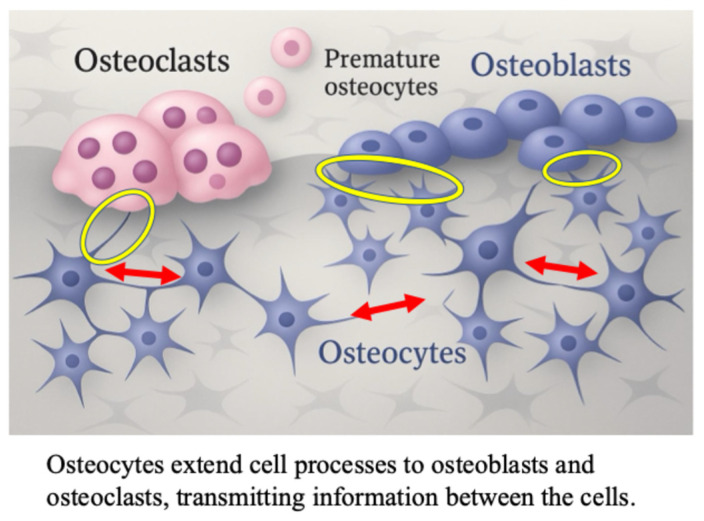
The role of osteocyte in osteoblast differentiation; cell-to-cell contact between osteocytes, osteoblasts and osteoclasts in bone tissue. The red arrows indicate osteocyte-osteocyte contact. The yellow circles indicate osteoblast-to-osteocyte contact.

Recently, the mechanism via which mechanical stress enhances bone formation has been elucidated [19,20,21,22,23]. Osteocytes are connected to adjacent osteoblasts via cellular processes extending into the lacunae and canaliculi, and control bone metabolism through intercellular communication [24,25,26,27,28,29] (Figure 1).

**Figure 2:**
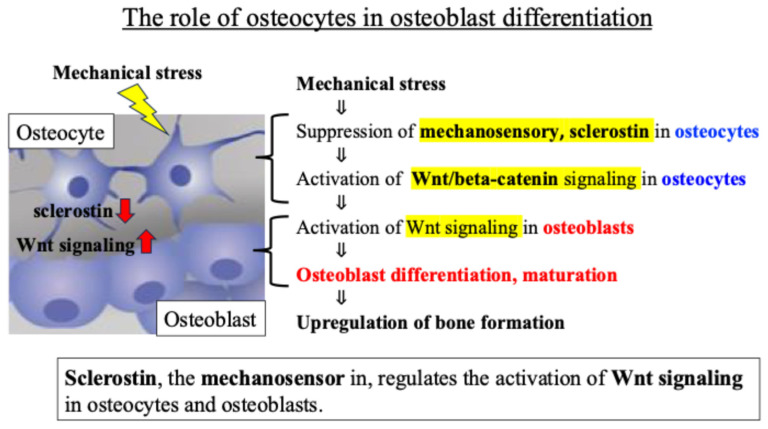
Intercellular communication network involving Wnt signaling between osteocytes and osteoblasts.

Osteocytes are known to regulate the activity of osteoblasts, which are responsible for bone formation, through the Wnt signaling pathway. The glycoprotein sclerostin produced by osteocytes negatively regulates osteoblast differentiation and bone formation by inhibiting Wnt signaling and activation [29]. Previous reports revealed that (i) in osteocytes sensing mechanical stress, the secretion and expression level of sclerostin decreases, (ii) the Wnt signal inhibited by sclerostin is activated, and (iii) as a result, osteoblast activity is enhanced, and bone formation is promoted [30,31] (Figure 2).

These findings indicate that an intercellular communication network, mainly involving Wnt signaling between osteocytes and osteoblasts, plays an important role in the response of bone tissue to mechanical stress (Figure 2).

## 3. Direct Activation of Osteoblasts by Mechanical Stress

Osteoblasts respond directly to mechanical stress, leading to improved cell activity and bone-forming ability [32,33,34]. However, it remains unknown how osteoblasts directly sense and respond to mechanical stress in the absence of the intercellular network formed by osteocytes; similarly, the related relationship between mechanical stress responses and bone-forming ability, and whether there are any defense mechanisms against pathological or excessive mechanical stress.

**Figure 3:**
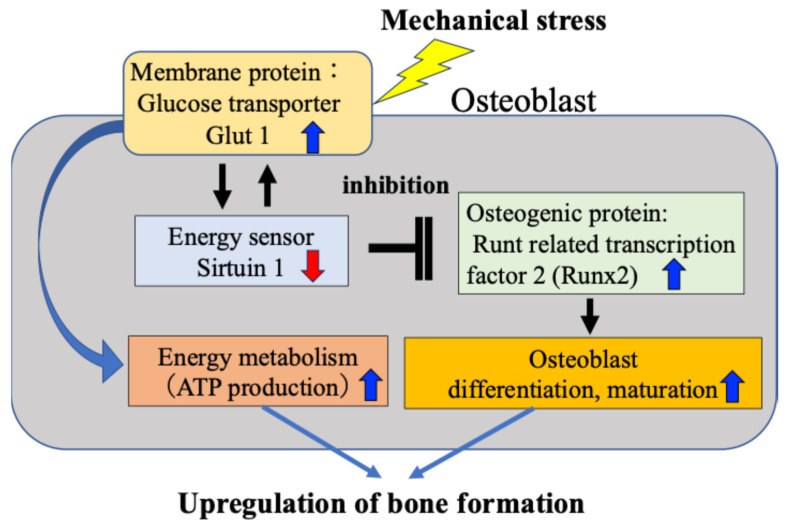
Direct activation of osteoblasts by mechanical stress. Mechanical stress directly induces ATP production, osteoblast activity and bone formation via the Glut1–SIRT1–Runx2 pathway in osteoblasts.

We have previously found that osteoblasts subject to mechanical stress increase intracellular glucose uptake through activation of Glut1 on the cell membrane, which in turn reduces activity of SIRT1 and consequently enhances expression of Runx2, which is negatively regulated by SIRT1 [15]. Furthermore, we confirmed that the Glut1–SIRT1 pathway–enhanced Runx2 pathway accelerates the differentiation and maturation of osteoblasts (osteocalcin and alkaline phosphatase activity) and their ability to synthesize bone matrix. We have reported for the first time that in response to mechanical stress, the osteogenic protein Runx2 is activated via the energy metabolism-regulating Glut1–SIRT1 signaling pathway, leading to osteoblast differentiation and bone formation (Figure 3).

Our findings revealed that the mechanism by which mechanical stress promotes bone formation in bone tissue is not only the previously reported mechanism mediated by the intercellular network between osteocytes and osteoblasts (Figure 1 and Figure 2), but also a response mechanism by which mechanical stress directly induces osteoblast differentiation and bone formation, in which SIRT1 activates Runx2 via Glut1 on the cell membrane (Figure 3). Although this is a tentative conclusion, we have discovered for the first time that Runx2 activity and bone formation are related to the mechanical stress response. Furthermore, our findings revealed that mechanism by which Glut1 on the osteoblast cell membrane is used as a mechanical stress sensor via SIRT1, without being involved in the intercellular network with osteocytes.

However, many aspects remain unclear regarding what initially senses mechanical stress in osteoblasts and how they respond via the Glut1–SIRT1–Runx2 pathway. Furthermore, the differences in the response of osteoblasts to physiological mechanical stress versus pathological excessive mechanical stress and the mechanisms of these different responses remain unclear. Clarifying these differences is expected to contribute to understanding the pathology of bone diseases and developing treatments. Further studies may reveal the existence of defense mechanisms in bone tissue against pathological excessive mechanical stress.

Based on the above, we are currently analyzing differences in the Glut1–SIRT1–Runx2 pathway in response to physiological and pathological mechanical stress, as well as the mechanisms underlying the regulation of osteoblast differentiation and bone formation through these pathways. We are also investigating the existence of defense mechanisms against excessive and pathological mechanical stress and related factors (paper in preparation). This is expected to provide clues to the etiology and pathology of bone diseases and bone-related systemic diseases, such as osteoporosis and age-related fractures, factors that cause patients to become bedridden, and to the development of new treatments.

Regarding the molecular mechanism of bone formation in response to mechanical stress, it is still unclear whether there is a mutual relationship between the Wnt-beta catenin signaling pathway in the osteocyte-osteoblast network and the Glut1-Sirt1-Runx2 pathway in osteoblast-only responses. To clarify these problems, it is necessary to identify the initial factors that sense mechanical stress and initiate signal transduction in both pathways, and to elucidate the details of these signal transduction pathways and their regulatory mechanisms, such as feedback mechanisms.

Recently, the role of subchondral bone in the development of osteochondral joint damage in OA has received increasing attention [35,36]. The functional conditions of articular cartilage and subchondral bone are tightly connected. As OA progresses, not only the articular cartilage layer but also the subchondral bone is damaged by mechanical stress. Mechanical stress on cartilage and subchondral bone adversely affects the mechanical environment and homeostatic balance of the entire joint, leading to the onset of OA. Subchondral bone deterioration has been widely recognized as a hallmark of OA.

As will be discussed in a later section, from the perspective of mechanical stress responses in subchondral bone tissue, the relationship between the Wnt-β-catenin signaling pathway and the Glut1-Sirt1-Runx2 pathway, as well as detailed analysis of these pathways, are important topics related to the mechanism of cartilage degeneration. In the near future, we will reveal whether there is a correlation between the two pathways, or whether each is an independent mechanism of bone metabolism.

## 4. Mechanical Stress and Articular Cartilage

Joint disorders caused by excessive sports and exercise are a major problem not only for the elderly but also for younger generations. In some cases, damage to chondrocytes and cartilage matrix progresses to an irreversible pathology of articular cartilage degeneration, and research into cartilage regenerative medicine is also underway [9,10]. However, preventive and therapeutic methods to suppress both joint pain and articular cartilage degeneration associated with the progression of OA symptoms are currently under development.

Obesity, weight bearing, and excessive mechanical stress on joints are known to cause degeneration and dysfunction of articular cartilage tissue, such as decreased chondrocyte activity and degeneration/destruction of the cartilage matrix, and are involved in the onset and pathology of OA [9,10,18,35]. However, much remains unknown about how chondrocytes respond to mechanical stress (mechanical stress sensing and response mechanisms). It is still unknown about the existence of defense mechanisms, and the relationship between the mechanisms of cartilage degeneration and stress responses. In order to develop effective preventive and therapeutic methods, we consider that it is necessary to elucidate the mechanisms of sensing and responding to mechanical stress, which are deeply involved in the etiology and pathology of articular cartilage degeneration.

## 5. Molecular Events Caused by Mechanical Stress in Articular Cartilage

We examined studies on (a) changes in the activity of DNA damage repair enzymes in chondrocytes and (b) the mechanism of chondrocyte energy metabolism regulation in response to mechanical stress [18,37,38,39]. We found evidence suggesting that (a) and (b) are closely related and together function as a defense mechanism against mechanical stress on articular cartilage, but that when exogenous stress can no longer be tolerated and this mutual defense function declines, cartilage degeneration progresses.

In this review we outline our findings on the interactions and networks between mechanisms that control the activity of DNA damage repair enzymes in chondrocytes and regulatory factors of cellular energy metabolism (glycolysis–citric acid cycle, and electron transport chain) in response to mechanical stress. We also discuss the basis for developing OA treatments through elucidating the mechanisms of mechanical stress response, defense, and cartilage degeneration.

### 5.1. Mechanical Stress-Induced DNA Damage in Chondrocytes

As is widely known, OA is characterized by a decline in joint tissue homeostasis, triggered by the disruption of the chondrocyte environment (degeneration of the cartilage matrix caused by external stresses such as mechanical stress, followed by enzymatic degradation, and further inflammation and immune responses), leading to the progressive degeneration and destruction of the joint over time [9,10,40,41]. Oxidative stress caused by reactive oxygen species (ROS; oxygen free radicals) that are produced in excess following mechanical stress on cartilage tissue has been attracting attention as an inducing factor for OA, which is the most prevalent of the musculoskeletal diseases [42,43,44,45,46].

All somatic cells produce ROS during the process of cellular respiration (mitochondrial respiratory chain function: energy production), which is the basis of life activities [47,48]. Aging and various external stresses can cause a decline in cellular respiratory chain function and a decrease in the activity of ROS-scavenging and antioxidant enzymes, leading to the excessive production and accumulation of ROS. This is closely related to the etiology and pathology of various age-related degenerative diseases and cancer [48,49].

**Figure 4:**
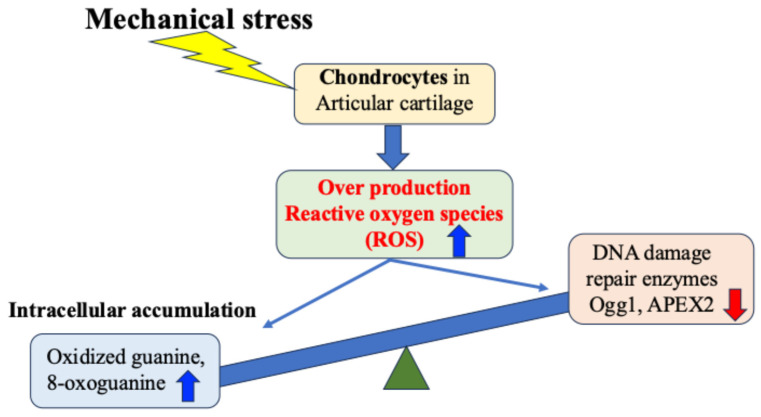
Mechanical stress-induced DNA damage in chondrocytes. Mechanical stress induces excessive production of reactive oxygen species, resulting in oxidative DNA damage and reduced repair enzyme activity.

Regarding OA, many studies both domestically and internationally have shown that mechanical stress on cartilage tissue causes the chronic production of ROS (oxidative stress) within cells and tissues, which in turn increases the production of inflammatory cytokines and causes secondary inflammation [18,35,50,51,52,53,54,55,56,57,58]. Attention has therefore been focused on the relationship between mechanical stress and oxidative stress control as a chondrocyte response in the etiology and pathology of OA [57,58] (Figure 4).

Green et al. experimentally demonstrated that mechanical stress on cartilage tissue induces excessive production of ROS from chondrocytes [52]. This excess of ROS induces chronic catabolism of chondrocytes and the cartilage matrix, disrupting articular cartilage homeostasis, and consequently causing cartilage degeneration and destruction [52]. However, how chondrocytes and cartilage tissue respond to mechanical stress, particularly the mechanisms of excessive production of ROS and cell damage caused by mechanical stress, are still under investigation.

Recently, the importance of DNA repair enzymes (such as Ogg1 and APEX2) has been highlighted as a cellular stress response mechanism [59,60]. Among the DNA damage caused by ROS in response to cellular stress, oxidation of guanine to 7,8-dihydro-8-oxoguanine enables it to pair with adenine instead of cytosine during DNA replication, resulting in a G:C-to-T:A point mutation, and such mutations are thought to be one of the causes of disease including cancer [60]. Decreased expression of DNA damage repair enzymes such as 8-oxoguanine DNA glycosylase1 (Ogg1) which specifically repairs oxidized guanine, has been reported in neurons of patients with amyotrophic lateral sclerosis and Alzheimer’s disease [61]. These findings suggest a link between these changes and the etiology/pathology of neurodegenerative diseases. These changes also suggest further involvement in bone and cartilage degenerative diseases other than neurological diseases.

We have found that expression of the DNA damage repair enzymes Ogg1 and APEX2 is highly correlated with the degree of degeneration of articular cartilage in OA [38,39]. Our in vitro research also demonstrated that expression of Ogg1 and APEX2 in response to OA inducers of cartilage degeneration and catabolism, such as interleukin-1β, was hardly observed in chondrocytes derived from normal cartilage, but was highly expressed in OA cartilage-derived chondrocytes [39]. In degenerated cartilage, DNA damage may result in increased expression of DNA oxidation products such as oxidized guanine, which in turn transiently increase the activity of Ogg1 and APEX2, which act as protective factors against DNA oxidative stress damage. However, it has been suggested that when the level of DNA damage exceeds the repair capacity of Ogg1 and APEX2, the DNA damage accumulates without being repaired. This may lead to cell death, a decline in tissue homeostasis, and joint degeneration (Figure 4).

Furthermore, we have discovered for the first time that SIRT1, which has nicotinamide adenine dinucleotide (NAD)-dependent deacetylase activity in OA chondrocytes and acts as a regulator of cellular energy metabolism, plays an important role in activating APEX2 and Ogg1 in chondrocytes [18]. Also, SIRT1 regulates Runx2, which is involved in hypertrophic cartilage formation and activation of the matrix-degrading enzyme matrix metalloproteinase (MMP)-13 for which Runx2 acts as a promoter, thereby regulating osteophyte formation and the production of cartilage matrix-degrading enzymes [18] These processes may be involved in the development of OA. Therefore, we next focused on the energy metabolism regulator, SIRT1, in chondrocytes, and outlined the relationship between the regulatory mechanism of energy metabolism and the activity of DNA damage repair enzymes.

### 5.2. Relationship Between Cellular Energy Metabolism Regulators and DNA Damage Repair Enzyme Activity in Chondrocytes

As mentioned above, the accumulation of ROS excessively produced from chondrocytes in response to mechanical stress such as obesity and weight-bearing is involved in the onset of OA, causing DNA damage and degeneration of the cartilage matrix in chondrocytes (Figure 4). Although it has been revealed that there is a close relationship between the mechanical stress response and the onset and progression of articular cartilage degeneration, the underlying mechanism and the existence of protective mechanisms remain largely unknown.

**Figure 5:**
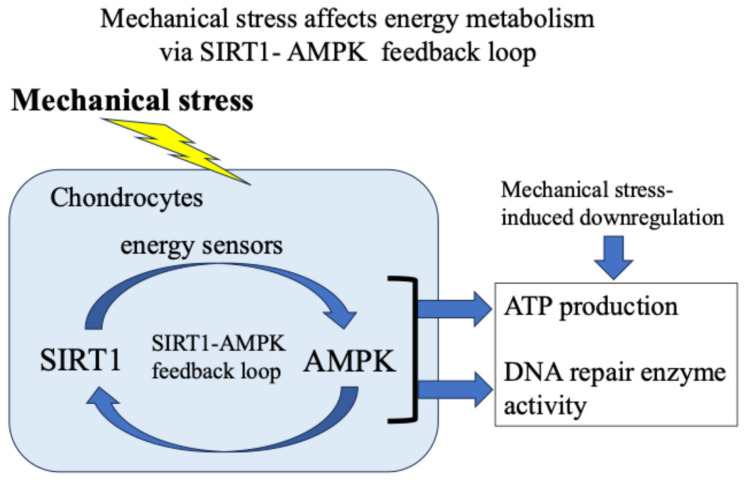
Mechanical stress-induced energy metabolism (ATP production) and DNA repair enzyme activity via a SIRT1–AMPK feedback loop.

Our and other reports have presented evidence supporting the idea that in response to exogenous stress such as weight bearing and inflammation, the energy metabolism of chondrocytes (glucose uptake and adenosine triphosphate/ATP production) temporarily increases [18,37,38,39]. Then, the activity of the citric acid cycle and electron transport chain in mitochondria increases, resulting in the production and leakage of excess ROS [48,49,50,51,52,53,54,55,56]. Furthermore, we found that 5′-AMP-activated protein kinase (AMPK), which acts as an intracellular energy sensor controlling ATP production, and SIRT1, which has NAD+-dependent deacetylase activity, form a mutual positive feedback mechanism that controls energy metabolism and alters responses to mechanical stress [18,62] (Figure 5).

**Figure 6:**
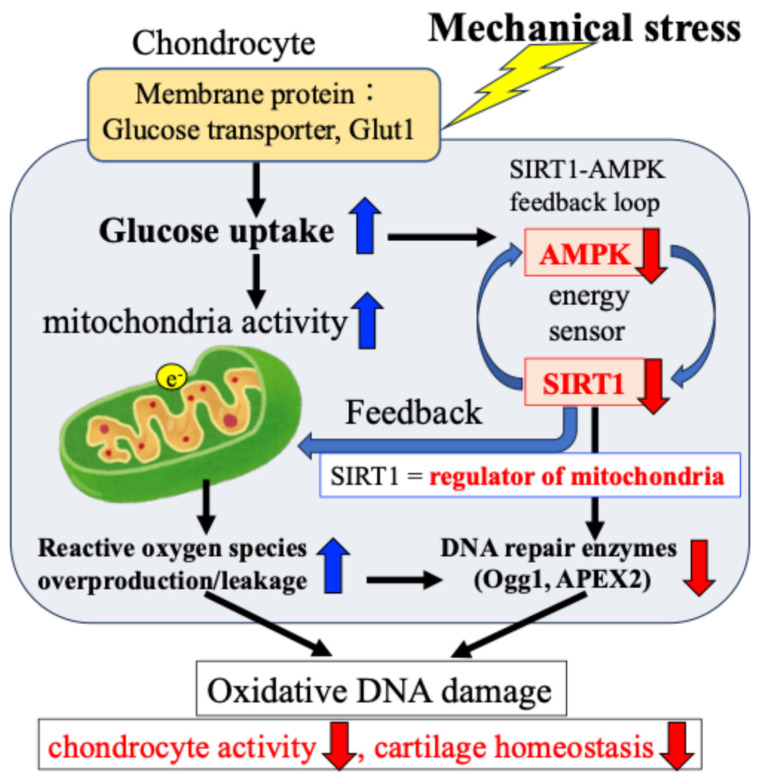
Mechanical stress-induced oxidative damage in chondrocytes. The e^−^ indicates electronic.

In addition, we confirmed that the AMPK–SIRT1 loop, which functions as a cellular energy sensor, regulates the activity of DNA damage repair enzymes. Specifically, as shown in Figure 6, in response to mechanical stress, (i) glucose uptake and ATP production in chondrocytes are transiently increased; (ii) the increased ATP production activates the citric acid cycle and electron transport chain in mitochondria, resulting in the production and leakage of excessive ROS (oxidative stress); (iii) the increased glucose uptake and ATP production are accompanied by a decrease in the activity of the energy sensor AMPK–SIRT1; and (iv) the decreased activity of this AMPK–SIRT1 feedback loop is associated with a decrease in the activity of Ogg1 and APEX2, leading to the persistence of DNA damage and a decrease in chondrocyte activity, resulting in disrupted cartilage homeostasis and cartilage catabolism in OA.

Cellular energy regulatory factors and DNA repair enzyme activity in response to mechanical stress are closely related to cartilage catabolism caused by ROS induced by mechanical stress. It is therefore possible that these mechanical stress response mechanisms may function as a type of defense mechanism. Next, based on this background, we summarized findings regarding candidate factors for the mechanical stress-sensing and response mechanisms and discussed the relationship between the mechanical stress response mechanism and the pathogenesis of OA.

### 5.3. Mechanical Stress Sensing and Response Mechanisms of Chondrocytes in OA

We have conducted extensive research into how chondrocytes sense and respond to mechanical stress, which is one of the causes of OA onset and pathogenesis, as well as the relationship between defense mechanisms against pathological and excessive stress and cartilage degeneration mechanisms.

**Figure 7:**
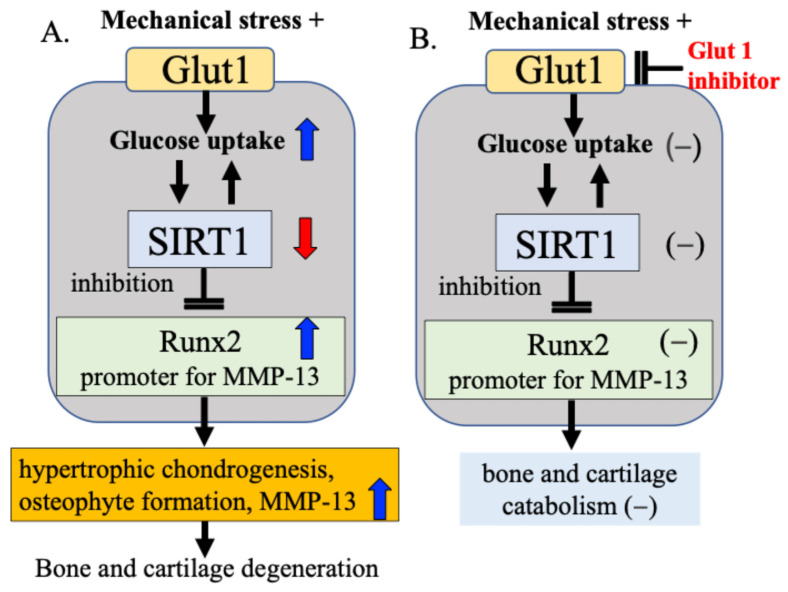
Mechanical stress sensing and response mechanisms in bone and cartilage metabolism. (**A**) Mechanical stress induces hypertrophic chondrogenesis, osteophyte formation, and increased MMP-13 production via the Gult1–SIRT1–Runx2 pathway in bone and cartilage metabolism. (**B**) When Glut1 function was inhibited using a Glut1 inhibitor, the expression of SIRT1 and Runx2 in response to mechanical stress was not observed, and changes in cellular activity in response to mechanical stress were also not observed.

Our research focuses on Glut1 as a cellular energy regulatory mechanism and SIRT1, which functions as a cellular energy metabolism sensor. Our results confirmed that bone and cartilage cells responding to mechanical stress alter the activity of Runx2 via the Glut1–SIRT1 signaling pathway, which controls cellular energy metabolism, leading to cartilage matrix degeneration and osteophyte formation [15]. We found that (1) mechanical stress increases the expression of Glut1 on the cell membrane, (2) increases glucose uptake into the cell through activation of Glut1, and in response, (3) decreases the activity of the cellular energy sensor and regulator SIRT1, and (4) increases the expression of Runx2, a hypertrophic chondrogenesis/osteogenesis transcription factor that is downregulated by SIRT1 [15]. These processes may change the cellular activity of chondrocytes and contribute to the degeneration of articular cartilage and the formation of osteophytes (Figure 7A). In our study, when Glut1 function was inhibited using a Glut1 inhibitor, the expression of SIRT1 and Runx2 in response to mechanical stress was not observed, and changes in cellular activity in response to mechanical stress were also not observed (Figure 7B), suggesting that Glut1 on the cell membrane is one of the factors that senses and responds to mechanical stress.

Based on these experimental results, we hypothesize that the Glut1–SIRT1 signaling pathway, a cellular energy regulatory factor, responds to mechanical stress on articular cartilage by controlling (i) DNA damage repair enzyme activity, (ii) cartilage catabolic factor Runx2 activity, and (iii) MMP-13 activity, a cartilage matrix-degrading enzyme for which Runx2 acts as a promoter. While this pathway acts to some extent as a defense mechanism, we speculate that when the defense mechanism is overwhelmed by excessive or pathological mechanical stress, and the mutual protective functions are impaired, bone and cartilage degeneration progresses (Figure 7).

## 6. Differences in Cellular Activity in Response to Physiological Levels of Mechanical Stress (Osteoblast vs. Chondrocyte)

Both bone and cartilage tissues are matrix-rich tissues, with 10–20% cellular components per volume [9,10,11]. While cartilage tissue contains only chondrocytes, bone tissue contains osteocytes, osteoblasts, and osteoclasts. Furthermore, cartilage is an avascular and nerveless tissue with no self-regenerative capacity [11,12,13]. On the other hand, bone is vascularized and has a rich remodeling capacity for bone resorption and bone formation. It is suggested that there may be similarities and differences between bone and cartilage in their responses to mechanical stress.

Our previous studies have revealed differences in the cellular activity of osteoblasts and chondrocytes in response to physiological levels of mechanical stress [15,37]. As shown in Table 1, at physiological loading levels that have been confirmed not to induce cell damage or cell death (apoptosis), osteoblast cellular activity was enhanced compared to non-loading conditions under both sustained and repeated loading, with repeated loading showing a stronger enhancement than sustained loading [15]. On the other hand, no change in chondrocyte activity was observed between non-loading and sustained loading. However, repeated loading conditions showed a tendency for chondrocyte activity to decrease compared to non-loading conditions [37] (Table 1).

Our studies suggest that osteoblast activity and bone formation capacity may be enhanced, while chondrocyte activity may be suppressed, in response to mechanical stress at physiological walking load levels. These findings indicate that chondrocytes in the articular cartilage layer and osteocytes, osteoblasts, and osteoclasts in the subchondral bone layer respond differently to mechanical stress in joint tissues.

Based on our findings, we hypothesized that chondrocytes in the articular cartilage layer and osteocytes, osteoblasts, and osteoclasts in the subchondral bone layer respond differently to mechanical stress in joint tissues.

## 7. Mechanisms of Mechanical Stress Response Other than the Wnt Pathway and GLT1-SIRT1-Runx2 Pathway in Bone and Cartilage Tissues

In addition to the Wnt signaling pathway, several mechano-transduction mechanisms have been reported in bone and cartilage tissues. Mechanical signals play critical roles in bone/cartilage homeostasis.

Recently, numerous reports have demonstrated that those Piezo proteins are important mechanosensitive channels in the bone microenvironment [63,64,65]. Piezo1 and Piezo2 form mechanosensitive cation channels [64,65,66]. It has been revealed that Piezo channels are mechanosensitive ion channels located in the plasma membrane and have been shown to function as important cellular mechanotransducers, converting mechanical stimuli into electrochemical signals [64,65]. In bone tissue, upon mechanical stimulation, these channels open, allowing cations to pass through the membrane, thereby facilitating cellular mechano-transduction and adapting to bone microenvironment [65,66]. Several studies have shown that Piezo1 is a mechanosensitive ion channel that allows osteoblasts to sense and respond to changes in mechanical loading [66,67].

In recent years, it has been revealed that ferroptosis occurs in chondrocytes of OA and plays an important role in the pathophysiological processes of OA [68,69,70]. Ferroptosis is a novel type of programmed cell death characterized by excess iron accumulation and lipid peroxidation [68]. Several reports demonstrated that nuclear factor E2-related factor 2 (Nrf2), glutathione peroxidase 4 (GPX4), solute carrier family 7 member 11 (SLC7A11), and p53 are key regulators of ferroptosis. It has been demonstrated that the GPX4, SLC7A11, and Nrf2 act as ferroptosis inhibitors, while p53 acts as a ferroptosis promoter and participates in the biological process of ferroptosis by regulating the antioxidant system and the formation of lipid reactive oxygen species [68,71]. More recently, Han J et al. demonstrated that mechanical stress activates the Nrf2 antioxidant system, inhibits the NF-kB p65 signaling pathway, and inhibits chondrocyte ferroptosis and cartilage degeneration by regulating P53, SLC7A11 and GPX4 [72]. These findings suggest that the NF-κB p65/GPX4 signaling pathway functions as a mechanosensing mechanism to suppress mechanical stress-induced ferroptosis in chondrocytes.

Transforming growth factor β (TGF-β) and bone morphometric proteins (BMPs) belong to the TGF-β superfamily and play important roles in osteoblast and chondrocyte differentiation, skeletal development, and osteochondral homeostasis [73,74]. Signaling by this protein family has been reported to not only specifically activate SMAD-dependent signaling and transcription, but also activate SMAD-independent signaling via MAPKs such as ERK and TAK1 [74]. These factors are also known to be closely involved in the mechanisms responding to mechanical stress, which also affects osteochondral tissue homeostasis [75]. Numerous reports have demonstrated that mechanical loading to the joint plays an important role in activating SMAD2/3 signaling in chondrocytes [74,75,76,77]. These observations suggest that SMAD2/3 signaling, activated by mechanical stress, plays a critical role in maintaining cartilage homeostasis. Considering the effects of SMAD3 signaling on cartilage, this mechanism is consistent with the observation that reduced joint loading contributes to cartilage degeneration, leading to the production of the proteolytic enzymes MMP8 and MMP13 [78]. It has been demonstrated that aged cartilage exhibits a significantly reduced capacity for mechanical loading-mediated SMAD2/3 signaling compared to younger cartilage, which may increase the risk of developing OA [79]. These findings also indicate the importance of mechanical loading-mediated SMAD2/3 signaling in maintaining cartilage homeostasis.

Mechanical loading has been also induced BMP levels in articular cartilage. It has been demonstrated that physiological exercise loading suppresses the progression of post-traumatic OA via increased expression of BMP2, BMP4, BMP6, and BMP receptor2 in vivo rat model [80]. Taken together, these studies show the impact of mechanical loading on TGFβ/BMP family signaling pathway.

## 8. Subchondral Bone Remodeling and Mechanical Stress

**Figure 8:**
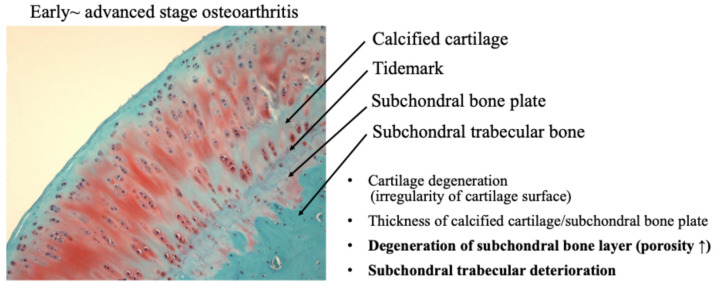
Osteochondral damage during the progression of osteoarthritis.

Recently, the role of subchondral bone in the development of osteochondral joint damage in OA has received increasing attention. It has been demonstrated that subchondral bone deterioration is widely recognized as a characteristic finding of OA [35,36]. Subchondral bone is a complex structure consisting of the subchondral plate and underlying spongy bone, which are biomechanically and biochemically connected to the overlying cartilage. OA subchondral bone is known to exhibit hypo-mineralization and abnormal bone metabolism, resulting in histopathological degeneration of the subchondral bone [35] (Figure 8).

A functional joint unit, consisting of articular cartilage and subchondral bone, may regulate the homeostasis and maintenance capacity of articular cartilage against mechanical stress during the progression of OA [81,82]. As OA progresses, not only the articular cartilage layer but also the subchondral bone is damaged by mechanical stress. Mechanical stress on cartilage and subchondral bone adversely affects the mechanical environment and homeostatic balance of the entire joint, leading to the onset of OA [36,82].

Crosstalk between articular cartilage and subchondral bone has been suggested to play a crucial role in the pathogenesis of osteoarthritis.

Previous studies have provided strong evidence that cartilage–bone cell interactions in OA joints result in bidirectional cellular phenotypic changes, leading to cartilage degeneration and sclerotic changes in the subchondral bone during OA progression [35,36,81,82]. It is well known that mechanical overloading disrupts subchondral bone remodeling before cartilage degeneration and the osteocytes in the subchondral bone are mainly responsible for mechanosensing. Osteocytes account for 90–95% of all skeletal cell population, approximately 20 times more than osteoblasts, and are considered the primary cell type responding to mechanical load [83]. More recently, it has been demonstrated that mechanical stress induces osteocytes in subchondral bone to secrete extracellular vesicles that downregulate chondrocyte metabolism [84]. Liu N. et al. demonstrated that osteocyte-derived extracellular vesicles mediate the bone-to-cartilage crosstalk and promote the progression of osteoarthritis [84]. It has been suggested that the miR-23b-3p in extracellular vesicles derived from osteocytes in the subchondral bone promoted cartilage catabolism and inhibits anabolism by inhibiting chondrocyte mitophagy. Also, they demonstrated that inhibition of miR-23b-3p in osteocytes suppresses cartilage degeneration and the progression of OA in experimental model mice. Their findings clearly indicate that extracellular vesicles secreted by subchondral osteocytes in response to mechanical stress mediate communication with chondrocytes and promote cartilage degeneration.

In addition to subchondral osteocytes under mechanical stress condition, subchondral osteoblasts also have a part in the bone-to-cartilage in OA progression. Several studies have demonstrated that exosomes derived from OA subchondral bone alter the transcriptional and bioenergetic properties of chondrocytes [36]. It is well known that exosomes are membrane-derived vesicles that have recently been recognized as important mediators of intercellular communication. Wu X et al. demonstrated that OA chondrocytes internalize exosomes derived from subchondral osteoblasts, induce the expression of catabolic genes, and downregulate the expression of chondrocyte-specific markers, a phenomenon commonly observed in OA cartilage [36]. RNA sequencing and miRNA profiling identified miR-210-5p, which is abundant in osteoblast exosomes derived from subchondral bone in OA, as an inducer of catabolic gene expression in articular chondrocytes. miR-210-5p has a role to suppress the oxygen consumption rate of chondrocytes and alter their bioenergetic state, a phenomenon commonly observed in OA pathology. These findings suggest that the altered crosstalk between OA cartilage and subchondral bone microenvironment is mediated by subchondral bone osteoblast-derived exosomes. In response to mechanical stress in OA, exosomes released from subchondral osteoblasts play an important role in the progression of cartilage degeneration, suggesting that they may be a potential target for therapeutic intervention in OA. The relationship between osteoblast-derived exosomes and the Glut1-SIRt1-Runx2 pathway in osteoblasts responding to mechanical stress has been still unclear, and we will plan to further elucidate this issue.

## 9. Conclusions

The cellular responses to physiological and pathological/excessive mechanical stress may differ among the cell populations that constitute the joint, particularly osteocytes, osteoblasts, and chondrocytes. As introduced in this review, there are still many aspects to be clarified regarding how the cells constituting bone and cartilage tissue sense and respond to stress (stress sensing and response factors) and whether there are defense reactions and mechanisms against pathological/excessive mechanical stress. Also, it remains unknown about the relationship between the mechanical stress sensing/response mechanisms and the mechanisms of bone and cartilage degeneration. Further research is required to clarify these issues. Therefore, we are currently focusing on mitochondrial function (energy metabolism) and autophagy, elucidating how mechanical stress affects autophagy as a cellular defense mechanism. Autophagy maintains intracellular energy homeostasis and protects cells against various stress conditions, such as inflammation and mechanical stress. Autophagy is an essential and conserved self-eating process carried out by cells to degrade intracellular components such as soluble proteins, aggregated proteins, organelles, macromolecular complexes, xenobiotics, and mitochondria. Cells have a function to degrade dysfunctional mitochondria through autophagy (self-eating), and when this function does not function properly, for example due to mechanical stress, mitochondrial activity decreases, which is thought to contribute to mechanical stress-induced diseases and aging.

Through these studies, we aim to develop new treatments for pathologies that alter cellular energy metabolism in response to mechanical stress, such as altered autophagy. We hope to gain insight into the development of new therapeutic methods through further clarification of these issues.

## Figures and Tables

**Table 1:** Differences in cellular activity in response to physiological levels of mechanical stress (Osteoblast vs. Chondrocyte).

	Non-Loading vs. Sustained Loading	Non-Loading vs. Repeated Loading
**Osteoblast energy metabolism**	**Non-loading < Sustained loading**	**Non-loading << Repeated loading**
**Chondrocyte energy metabolism**	**Non-loading** **≒** **Sustained load**	**Non-loading >> Repeated loading**
Under physiological stress confirmed not to induce cell damage or apoptosis (non-pathological stress level)		
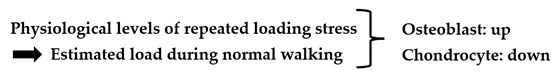		

## Data Availability

No new data were created or analyzed in this study. Data sharing is not applicable to this article.

## References

[1] Kanis, J.A. on behalf of the World Health Organization Scientific Group (2007). Assessment of Osteoporosis at the Primary Health-care Level.

[2] O’Flynn N. (2012). Risk assessment of fragility fracture: NICE guideline. Br. J. Gen. Pract..

[3] Cooper C., Atkinson E.J., Jacobsen S.J., O’Fallon W.M., Melton L.J. (1993). Population-based study of survival after osteoporotic fractures. Am. J. Epidemiol..

[4] Salari N., Ghasemi H., Mohammadi L., Behzadi M.H., Rabieenia E., Shohaimi S., Mohammadi M. (2021). The global prevalence of osteoporosis in the world: A comprehensive systematic review and meta-analysis. J. Orthop. Surg. Res..

[5] Bliuc D., Nguyen N.D., Milch V.E., Nguyen T.V., Eisman J.A., Center J.R. (2009). Mortality risk associated with low-trauma osteoporotic fracture and subsequent fracture in men and women. J. Am. Med. Assoc..

[6] Takusari E., Sakata K., Hashimoto T., Fukushima Y., Nakamura T., Orimo H. (2020). Trends in hip fracture incidence in Japan: Estimates based on nationwide hip fracture surveys from 1992 to 2017. JBMR Plus.

[7] Hagino H., Endo N., Harada A., Iwamoto J., Mashiba T., Mori S., Ohtori S., Sakai A., Takada J., Yamamoto T. (2017). Survey of hip fractures in Japan: Recent trends in prevalence and treatment. J. Orthop. Sci..

[8] Abe K., Inage K., Yoshimura K., Sato D., Yamashita K., Yamashita M., Sasaki T., Yamaoka A., Shiga Y., Eguchi Y. (2024). Deaths caused by osteoporotic fractures in Japan: An epidemiologicalstudy. J. Orthop. Sci..

[9] Bannuru R.R., Osani M.C., Vaysbrot E.E., Arden N.K., Bennell K., Bierma-Zeinstra S.M.A., Kraus V.B., Lohmander L.S., Abbott J.H., Bhandari M. (2019). OARSI guidelines for the non-surgical management of knee, hip, and polyarticular osteoarthritis. Osteoarthr. Cartil..

[10] Macri E.M., Selles R.W., Stefanik J.J., Reijman M. (2023). OARSI year in review 2023: Rehabilitation and outcomes. Osteoarthr. Cartil..

[11] Yoshimura N. (2011). Epidemiology of osteoarthritis in Japan: The ROAD study. Clin. Calcium.

[12] Yoshimura N., Muraki S., Iidaka T., Oka H., Horii C., Kawaguchi H., Akune T., Nakamura K., Tanaka S. (2019). Prevalence and co-existence of locomotive syndrome, sarcopenia, and frailty: The third survey of Research on Osteoarthritis/Osteoporosis Against Disability (ROAD) study. J. Bone Miner. Metab..

[13] Yoshimura N., Iidaka T., Horii C., Mure K., Muraki S., Oka H., Kawaguchi H., Akune T., Ishibashi H., Ohe T. (2022). Epidemiology of locomotive syndrome using updated clinical decision limits: 6-year follow-ups of the ROAD study. J. Bone Miner. Metab..

[14] Hiligsmann M., Cooper C., Guillemin F., Hochberg M.C., Tugwell P., Arden N., Berenbaum F., Boers M., Boonen A., Branco J.C. (2014). A reference case for economic evaluations in osteoarthritis: An expert consensus article from the European Society for Clinical and Economic Aspects of Osteoporosis and Osteoarthritis (ESCEO). Semin. Arthritis Rheum..

[15] Somemura S., Kumai T., Yatabe K., Sasaki C., Fujiya H., Niki H., Yudoh K. (2021). Physiologic Mechanical Stress Directly Induces Bone Formation by Activating Glucose Transporter 1 (Glut 1) in Osteoblasts, Inducing Signaling via NAD+-Dependent Deacetylase (Sirtuin 1) and Runt-Related Transcription Factor 2 (Runx2). Int. J. Mol. Sci..

[16] Zaidi M., Zaidi S., Yuen T. (2024). Understanding osteokine biology. Cell Metab..

[17] Yang L., Chen H., Yang C., Hu Z., Jiang Z., Meng S., Liu R., Huang L., Yang K. (2024). Research progress on the regulatory mechanism of integrin-mediated mechanical stress in cells involved in bone metabolism. J. Cell. Mol. Med..

[18] Terauchi K., Kobayashi H., Yatabe K., Yui N., Fujiya H., Niki H., Musha H., Yudoh K. (2016). The NAD-Dependent Deacetylase Sirtuin-1 Regulates the Expression of Osteogenic Transcriptional Activator Runt-Related Transcription Factor 2 (Runx2) and Production of Matrix Metalloproteinase (MMP)-13 in Chondrocytes in Osteoarthritis. Int. J. Mol. Sci..

[19] Bonewald L.F., Johnson M.L. (2008). Osteocytes, mechanosensing and Wnt signaling. Bone.

[20] Yuan Y., Zhang L., Tong X., Zhang M., Zhao Y., Guo J., Lei L., Chen X., Tickner J., Xu J. (2017). Mechanical Stress Regulates Bone Metabolism Through MicroRNAs. J. Cell. Physiol..

[21] Hu W., Guo Z., Tang W., Long J. (2024). Mechanoresponsive regulation of tissue regeneration during distraction osteogenesis. FASEB J..

[22] Liang W., Wei T., Hu L., Chen M., Tong L., Zhou W., Duan X., Zhao X., Zhou W., Jiang Q. (2024). An integrated multi-omics analysis reveals osteokines involved in global regulation. Cell Metab..

[23] Burgers T.A., Williams B.O. (2013). Regulation of Wnt/beta-catenin signaling within and from osteocytes. Bone.

[24] Lin C., Jiang X., Dai Z., Guo X., Weng T., Wang J., Li Y., Feng G., Gao X., He L. (2009). Sclerostin mediates bone response to mechanical unloading through antagonizing Wnt/beta-catenin signaling. J. Bone Miner. Res..

[25] Sapir-Koren R., Livshits G. (2014). Osteocyte control of bone remodeling: Is sclerostin a key molecular coordinator of the balanced bone resorption–formation cycles?. Osteoporos. Int..

[26] Galea G.L., Lanyon L.E., Price J.S. (2016). Sclerostin’s role in bone’s adaptive response to mechanical loading. Bone.

[27] Uda Y., Azab E., Sun N., Shi C., Pajevic P.D. (2017). Osteocyte mechanobiology. Curr. Osteoporos. Rep..

[28] Hinton P.V., Rackard S.M., Kennedy O.D. (2018). In vivo osteocyte mechanotransduction: Recent developments and future directions. Curr. Osteoporos. Rep..

[29] Fu R., Feng Y., Liu Y., Yang H. (2021). Mechanical regulation of bone regeneration during distraction osteogenesis. Med. Novel Technol. Dev..

[30] Spatz J., Wein M.N., Gooi J., Qu Y., Garr J.L., Liu S., Barry K.J., Uda Y., Lai F., Dedic C. (2015). The wnt inhibitor sclerostin is up-regulated by mechanical unloading in osteocytes in Vitro. J. Biol. Chem..

[31] Chen X., Guo J., Yuan Y., Sun Z., Chen B., Tong X., Zhang L., Shen C., Zou J. (2017). Cyclic compression stimulates osteoblast differentiation via activation of the Wnt/β-catenin signaling pathway. Mol. Med. Rep..

[32] Liu C., Feng N., Wang Z., Zheng K., Xie Y., Wang H., Long H., Peng S. (2024). Foxk1 promotes bone formation through inducing aerobic glycolysis. Cell Death Differ..

[33] Jin X., Sun X., Ma X., Qin Z., Gao X., Kang X., Li H., Sun H. (2024). SIRT1 maintains bone homeostasis by regulating osteoblast glycolysis through GOT1. Cell. Mol. Life Sci..

[34] Zhu Q., Fu Y., Cui C.P., Ding Y., Deng Z., Ning C., Hu F., Qiu C., Yu B., Zhou X. (2023). OTUB1 promotes osteoblastic bone formation through stabilizing FGFR2. Signal Transduct. Target. Ther..

[35] Pan J., Wang B., Li W., Zhou X., Scherr T., Yang Y., Price C., Wang L. (2012). Elevated cross-talk between subchondral bone and cartilage in osteoarthritic joints. Bone.

[36] Wu X., Crawford R., Xiao Y., Mao X., Prasadam I. (2021). Osteoarthritic Subchondral Bone Release Exosomes That Promote Cartilage Degeneration. Cells.

[37] Takemoto M., Sugishita Y., Takahashi-Suzuki Y., Fujiya H., Niki H., Yudoh K. (2025). Repetitive Compressive Loading Downregulates Mitochondria Function and Upregulates the Cartilage Matrix Degrading Enzyme MMP-13 Through the Coactivation of NAD-Dependent Sirtuin 1 and Runx2 in Osteoarthritic Chondrocytes. Int. J. Mol. Sci..

[38] Yui N., Yoshioka H., Fujiya H., Musha H., Beppu M., Karasawa R., Yudoh K. (2014). The DNA repair enzyme apurinic/apyrimidinic endonuclease (Apex nuclease) 2 has the potential to protect against down-regulation of chondrocyte activity in osteoarthritis. Int. J. Mol. Sci..

[39] Yui N., Yudoh K., Fujiya H., Musha H. (2016). Mechanical and oxidative stress in osteoarthritis. J. Phys. Fitness.

[40] Nedunchezhiyan U., Varughese I., Sun A.R., Wu X., Crawford R., Prasadam I. (2022). Obesity, Inflammation, and Immune System in Osteoarthritis. Front. Immunol..

[41] Vincent T.L., Miller R.E. (2024). Molecular pathogenesis of OA pain: Past, present, and future. Osteoarthr. Cartil..

[42] Coryell P.R., Diekman B.O., Loeser R.F. (2021). Mechanisms and therapeutic implications of cellular senescence in osteoarthritis. Nat. Rev. Rheumatol..

[43] Liu L., Luo P., Yang M., Wang J., Hou W., Xu P. (2022). The role of oxidative stress in the development of knee osteoarthritis: A comprehensive research review. Front. Mol. Biosci..

[44] Riegger J., Schoppa A., Ruths L., Haffner-Luntzer M., Ignatius A. (2023). Oxidative stress as a key modulator of cell fate decision in osteoarthritis and osteoporosis: A narrative review. Cell. Mol. Biol. Lett..

[45] Zeng Y., Yu S., Lu L., Zhang J., Xu C. (2024). Ginger-derived nanovesicles attenuate osteoarthritis progression by inhibiting oxidative stress via the Nrf2 pathway. Nanomedicine.

[46] Liu C., Wang X., Zhang Y., Ge H., Chang Q., Zhou Z. (2025). beta-Sitosterol preconditioning enhances the resistance of BMSCs and chondrocyte to oxidative stress and promotes cartilage repair in osteoarthritis. Stem Cell Res. Ther..

[47] Kuznetsov A.V., Margreiter R., Ausserlechner M.J., Hagenbuchner J. (2022). The Complex Interplay between Mitochondria, ROS and Entire Cellular Metabolism. Antioxidants.

[48] Zhang J., Simpson C.M., Berner J., Chong H.B., Fang J., Ordulu Z., Weiss-Sadan T., Possemato A.P., Harry S., Takahashi M. (2023). Systematic identification of anticancer drug targets reveals a nucleus-to-mitochondria ROS-sensing pathway. Cell.

[49] Casati S.R., Cervia D., Roux-Biejat P., Moscheni C., Perrotta C., De Palma C. (2024). Mitochondria and Reactive Oxygen Species: The Therapeutic Balance of Powers for Duchenne Muscular Dystrophy. Cells.

[50] Kurz B., Lemke A., Kehn M., Domm C., Patwari P., Frank E.H., Grodzinsky A.J., Schünke M. (2004). Influence of tissue maturation and antioxidants on the apoptotic response of articular cartilage after injurious compression. Arthritis Rheum..

[51] Kurz B., Lemke A.K., Fay J., Pufe T., Grodzinsky A.J., Schunke M. (2005). Pathomechanisms of cartilage destruction by mechanical injury. Ann. Anat..

[52] Green D.M., Noble P.C., Ahuero J.S., Birdsall H.H. (2006). Cellular events leading to chondrocyte death after cartilage impact injury. Arthritis Rheum..

[53] Wolff K.J., Ramakrishnan P.S., Brouillette M.J., Journot B.J., McKinley T.O., Buckwalter J.A., Martin J.A. (2013). Mechanical stress and ATP synthesis are coupled by mitochondrial oxidants in articular cartilage. J. Orthop. Res..

[54] Chang S.H., Mori D., Kobayashi H., Mori Y., Nakamoto H., Okada K., Taniguchi Y., Sugita S., Yano F., Chung U.-I. (2019). Excessive mechanical loading promotes osteoarthritis through the gremlin-1–NF-κB pathway. Nat. Commun..

[55] He Y., Makarczyk M.J., Lin H. (2020). Role of mitochondria in mediating chondrocyte response to mechanical stimuli. Life Sci..

[56] Takeda Y., Niki Y., Fukuhara Y., Fukuda Y., Udagawa K., Shimoda M., Kikuchi T., Kobayashi S., Harato K., Miyamoto T. (2021). Compressive mechanical stress enhances susceptibility to interleukin-1 by increasing interleukin-1 receptor expression in 3D-cultured ATDC5 cells. BMC Musculoskelet. Disord..

[57] Shao Y., Zhang H., Guan H., Wu C., Qi W., Yang L., Yin J., Zhang H., Liu L., Lu Y. (2024). PDZK1 protects against mechanical overload-induced chondrocyte senescence and osteoarthritis by targeting mitochondrial function. Bone Res..

[58] Momin A., Perrotti S., Waldman S.D. (2024). The role of mitochondrial reactive oxygen species in chondrocyte mechanotransduction. J. Orthop. Res..

[59] Nakabeppu Y., Tsuchimoto D., Furuichi M., Sakumi K. (2004). The defense mechanisms in mammalian cells against oxidative damage in nucleic acids and their involvement in the suppression of mutagenesis and cell death. Free Radic. Res..

[60] Nakabeppu Y., Tsuchimoto D., Ichinoe A., Ohno M., Ide Y., Hirano S., Yoshimura D., Tominaga Y., Furuichi M., Sakumi K. (2004). Biological significance of the defense mechanisms against oxidative damage in nucleic acids caused by reactive oxygen species: From mitochondria to nuclei. Ann. N.Y. Acad. Sci..

[61] Nunomura A., Moreira P.I., Castellani R.J., Lee H.G., Zhu X., Smith M.A., Perry G. (2012). Oxidative damage to RNA in aging and neurodegenerative disorders. Neurotox. Res..

[62] Kobayashi H., Terauchi K., Yui N., Yatabe K., Kamada T., Fujiya H., Niki H., Musha H., Yudoh K. (2017). The Nicotinamide Adenine Dinucleotide (NAD)-Dependent Deacetylase Sirtuin-1 Regulates Chondrocyte Energy Metabolism through the Modulation of Adenosine Monophosphate-Activated Protein Kinase (AMPK) in Osteoarthritis (OA). J. Arthritis.

[63] Poole K., Herget R., Lapatsina L., Ngo H.D., Lewin G.R. (2014). Tuning piezo ion channels to detect molecular-scale movements relevant for fine touch. Nat. Commun..

[64] Zhao Q., Wu K., Geng J., Chi S., Wang Y., Zhi P., Zhang M., Xiao B. (2016). Ion permeation and mechanotransduction mechanisms of mechanosensitive piezo channels. Neuron.

[65] Li X., Han L., Nookaew I., Mannen E., Silva M.J., Almeida M., Xiong J. (2019). Stimulation of Piezo1 by mechanical signals promotes bone anabolism. eLife.

[66] Yuan G., Xiong Z., Ke X., Wang G., Liu X., Li Z. (2025). Exploring the Multifactorial Regulation of PIEZO1 in Chondrocytes: Mechanisms and Implications. Int. J. Med. Sci..

[67] Zhou T., Gao B., Fan Y., Liu Y., Feng S., Cong Q., Zhang X., Zhou Y., Yadav P.S., Lin J. (2020). Piezo1/2 mediate mechanotransduction essential for bone formation through concerted activation of NFAT-YAP1-ß-Catenin. eLife.

[68] Li J., Cao F., Yin H.-L., Huang Z.-J., Lin Z.-T., Mao N., Sun B., Wang G. (2020). Ferroptosis: Past, present and future. Cell Death Dis..

[69] Lv Z., Han J., Li J., Guo H., Fei Y., Sun Z., Dong J., Wang M., Fan C., Li W. (2022). Single cell RNA-seq analysis identifies ferroptotic chondrocyte cluster and reveals TRPV1 as an anti-ferroptotic target in osteoarthritis. EBioMedicine.

[70] Miao Y., Chen Y., Xue F., Liu K., Zhu B., Gao J., Yin J., Zhang C., Li G. (2022). Contribution of ferroptosis and GPX4’s dual functions to osteoarthritis progression. EBioMedicine.

[71] Tang D., Chen X., Kang R., Kroemer G. (2021). Ferroptosis: Molecular mechanisms and health implications. Cell Res..

[72] Han J., Zhan L.-N., Huang Y., Guo S., Zhou X., Kapilevich L., Wang Z., Ning K., Sun M., Zhang X.-a. (2024). Moderate mechanical stress suppresses chondrocyte ferroptosis in osteoarthritis by regulating NF-κB p65/GPX4 signaling pathway. Sci. Rep..

[73] Thielen N.G., van der Kraan P.M., van Caam A.P. (2019). TGFβ/BMP Signaling Pathway in Cartilage Homeostasis. Cells.

[74] Wu M., Wu S., Chen W., Li Y.-P. (2024). The roles and regulatory mechanisms of TGF-β and BMP signaling in bone and cartilage development, homeostasis and disease. Cell Res..

[75] Madej W., van Caam A., Blaney Davidson E.N., van der Kraan P.M., Buma P. (2014). Physiological and excessive mechanical compression of articular cartilage activates Smad2/3P signaling. Osteoarthr. Cartil..

[76] Responte D.J., Lee J.K., Hu J.C., Athanasiou K.A. (2012). Biomechanics-driven chondrogenesis: From embryo to adult. FASEB J..

[77] Nam J., Perera P., Rath B., Agarwal S. (2013). Dynamic regulation of bone morphogenetic proteins in engineered osteochondral constructs by biomechanical stimulation. Tissue Eng. Part A.

[78] Nomura M., Sakitani N., Iwasawa H., Kohara Y., Takano S., Wakimoto Y., Kuroki H., Moriyama H. (2017). Thinning of articular cartilage after joint unloading or immobilization. An experimental investigation of the pathogenesis in mice. Osteoarthr. Cartil..

[79] Madej W., van Caam A., Davidson E.N., Hannink G., Buma P., van der Kraan P.M. (2016). Ageing is associated with reduction of mechanically-induced activation of Smad2/3P signaling in articular cartilage. Osteoarthr. Cartil..

[80] Iijima H., Ito A., Nagai M., Tajino J., Yamaguchi S., Kiyan W., Nakahata A., Zhang J., Wang T., Aoyama T. (2017). Physiological exercise loading suppresses post-traumatic osteoarthritis progression via an increase in bone morphogenetic proteins expression in an experimental rat knee model. Osteoarthr. Cartil..

[81] Prasadam I., Van Gennip S., Friis T., Shi W., Crawford R., Xiao Y. (2010). ERK-1/2 and p38 in the regulation of hypertrophic changes of normal articular cartilage chondrocytes induced by osteoarthritic subchondral osteoblasts. Arthritis Rheum..

[82] Prasadam I., Crawford R., Xiao Y. (2012). Aggravation of ADAMTS and Matrix Metalloproteinase Production and Role of ERK1/2 Pathway in the Interaction of Osteoarthritic Subchondral Bone Osteoblasts and Articular Cartilage Chondrocytes—Possible Pathogenic Role in Osteoarthritis. J. Rheumatol..

[83] Jaiprakash A., Prasadam I., Feng J.Q., Liu Y., Crawford R., Xiao Y. (2012). Phenotypic characterization of osteoarthritic osteocytes from the sclerotic zones: A possible pathological role in subchondral bone sclerosis. Int. J. Biol. Sci..

[84] Liu N., Ma Y., Gong W., Shao X., Shi T., Li L., Wu W., Chen X., Shi Y., Zhang P. (2025). Osteocyte-derived extracellular vesicles mediate the bone-to-cartilage crosstalk and promote osteoarthritis progression. Nat. Commun..

